# Learning curve for endoscopic tympanoplasty type I: comparison of endoscopic-native and microscopically-trained surgeons

**DOI:** 10.1007/s00405-020-06293-0

**Published:** 2020-08-27

**Authors:** Eduardo Machado Rossi Monteiro, Sven Beckmann, Maísa Mendes Pedrosa, Till Siggemann, Sarah Maciel Augusta Morato, Lukas Anschuetz

**Affiliations:** 1 Department of Otorhinolaryngology, Hospital Felicio Rocho, Belo Horizonte, Brasil; 2 Department of Otorhinolaryngology, Head and Neck Surgery, Inselspital, University Hospital and University of Bern, Freiburgstrasse 16, 3010 Bern, Switzerland

**Keywords:** Endoscopic ear surgery, Endoscopic tympanoplasty, Tympanoplasty type I, Learning curve

## Abstract

**Purpose:**

Type I tympanoplasty is one of the first operations to be performed by ear surgeons in training and is increasingly performed using the endoscopic technique. The aim of the present study is to assess and compare the learning curve for type I tympanoplasties between a microscopically trained and endoscopic native ear surgeon. We hypothesize comparable learning curves between the two surgeons regardless of previous microscopic experience.

**Methods:**

Retrospective analysis and comparison of the 25 first consecutive cases of type I tympanoplasty performed by a microscopically trained ear surgeon (MTES) and a native endoscopic ear surgeon (NEES).

**Results:**

Mean duration of surgery in MTES and NEES groups was 54 ± 12.3 min and 55.6 ± 17.5 min, respectively. Both surgeons achieved a reduction of the surgery duration over time with statistically significant reduction from the first five cases to the last five cases in both groups. Graft intake rate was 92% after 3 months. Preoperative and postoperative PTA revealed a mean improvement of air bone gap (ABG) of 11.5 ± 7.1 dB HL in MTES group versus 9.3 ± 8.5 dB HL in NEES group, whereby the difference between the two groups was not statistically significant.

**Conclusion:**

Endoscopic type I tympanoplasty shows comparable results and learning curves in two beginning endoscopic ear surgeons independent of the previous microscopic experience. We recommend if available the parallel learning of both techniques.

## Introduction

With the spread of endoscopic ear surgery (EES) in the last three decades, the endoscopic approach to type I tympanoplasty has gained increasing attention. The first endoscopic transcanal myringoplasty in humans was described in 1992 by El Guindy et al. [1], followed by the first cohort of endoscopic tympanoplasties in 1999, demonstrating similar results compared to conventional microscopic approaches [2]. More recently, systematic reviews and meta-analyses found similar functional, hearing and safety results for endoscopic compared to microscopic tympanoplasty. However, a lower rate of canaloplasty, fewer wound complications, shorter operative times and higher cosmetic satisfaction was described for EES [3–5]. Furthermore, patients undergoing the intervention using the endoscopic approach, seem to use fewer medical resources as patients with microscopic tympanoplasty due to the shorter time spent in the operating room [6]. This might lead to an increasing implementation of endoscopic tympanoplasty in the future, although a longer learning curve for the endoscopic approach is presumed. However, little evidence is available regarding this topic. It has been shown, that favorable and equivalent results can be achieved during the transition from microscopic to endoscopic tympanoplasty and teaching it to residents [7].

Since type I tympanoplasty may be considered an ideal operation to learn middle ear surgery, the question arises whether the trainee should be first taught using the traditional microscopic or the endoscopic technique. Both techniques have their inherent advantages and challenges. These questions may have an important impact on the way we learn and teach middle ear surgeries as well as considerable socio-economic consequences. Therefore, we aim to further investigate differences in learning curves regarding endoscopic type I tympanoplasties in surgeons of different educational levels. This comparison may give some evidence about the most suitable sequence of techniques used in the learning of type I tympanoplasties. To this end the intraoperative performance and postoperative results regarding the first consecutive type I tympanoplasties of an experienced microscopic to an endoscopic-native surgeon were investigated. We hypothesize comparable learning curves and outcomes in tympanoplasty type I, regardless of the previous microscopic experience.

## Patients and methods

### Patients

In this study 50 cases of tympanoplasty type I were retrospectively enrolled. The local institutional review boards approved this study (reference number: KEK Bern 2019-00555 and Plataforma Brasil 87370218.3.0000.5125). To answer the study question, the first 25 consecutive endoscopic type I tympanoplasties of a microscopically trained ear surgeon (E.R., 8 years of experience in microscopic ear surgery) were compared to the first 25 cases of an endoscopic-native ear surgeon (L.A.). All patients suffered from chronic otitis media with central perforations of all sizes. Patients with cholesteatoma, otomastoiditis or requiring any kind of ossiculoplasty were excluded from the present study. The intraoperative records and the patient’s charts were collected and analyzed. Follow-up assessments were performed 4 weeks and 3 months after surgery including pure tone audiometry (PTA). Pre- and post-operative PTA were analyzed regarding air-conduction thresholds (ACT) and bone-conduction thresholds (BCT) at 500, 1000, 2000, 3000 and 4000 Hz as well as ACT for 6000 and 8000 Hz. Five audiograms from the MTES group for frequencies of 6000 and 8000 Hz were not available. The air–bone gap (ABG) and its post-operative evolution were calculated. Full ABG closure was defined as mean ABG < 10 dB.

### Surgical technique

For all surgeries, both surgeons used the underlay technique. After excision of the perforation margins, a tympano-meatal flap was elevated and the mobility of the ossicular chain tested. Grafts used were fascia or full thickness tragal cartilage after removal of perichondrium on both sides. The estimated average thickness was around 1 mm. The cartilage was inserted in underlay technique and the perichondrium used to reinforce the reconstruction where needed. A absorbable packing inside the middle ear with Gelfoam® supported the reconstruction. Afterwards the tympano-meatal flap was repositioned and an absorbable packing placed inside the external auditory canal.

### Statistical analysis

The assessed endpoints were duration of surgery, graft intake rate (GIR), intra- and post-operative complications and hearing outcomes. Patients were divided into two groups: the first 25 consecutive operations of the microscopically trained ear surgeon (MTES) were compared with the first 25 consecutive operations of the native endoscopic ear surgeon (NEES). Descriptive and comparative statistical analysis was performed with students *t*-test between both groups in GraphPad Prism 8. A statistically significant difference was assumed for a two-tailed alpha < 0.05.

## Results

A total of 50 endoscopic type I tympanoplasty were analyzed. The first 25 consecutive cases of both surgeons (MTES and NEES) were enrolled. All operations were performed without need for conversion to a microscopic approach and no canaloplasty was required in any case. Mean age (± standard deviation) was comparable between both groups and was assessed in the MTES group 40.8 years (± 17.5 years) compared to 40.2 years (± 21.6 years) in the NEES group. The MTES group included 10 women versus 7 women in the NEES group. Patient characteristics of both groups are summarized in Table 1. There was no occurrence of postoperative facial palsy or sensorineural hearing loss (SNHL) after endoscopic tympanoplasty type I throughout the cohort.

**Table 1:** Patient characteristics

	MTES	NEES
Age ± SD (years)	40.8 ± 17.5	40.2 ± 21.6
Sex		
Female	10 (40%)	7 (28%)
Side		
Left	14 (56%)	12 (48%)
Perforation size		
< 25%	12 (48%)	10 (40%)
25–50%	12 (48%)	10 (40%)
50–75%	1 (4%)	1 (4%)
> 75%	0 (0%)	4 (16%)
Mean preoperative ABG ± SD (dB)	21.1 ± 6.9	17.4 ± 8.3

### Comparison of learning curve and surgical results

**Fig. 1:**
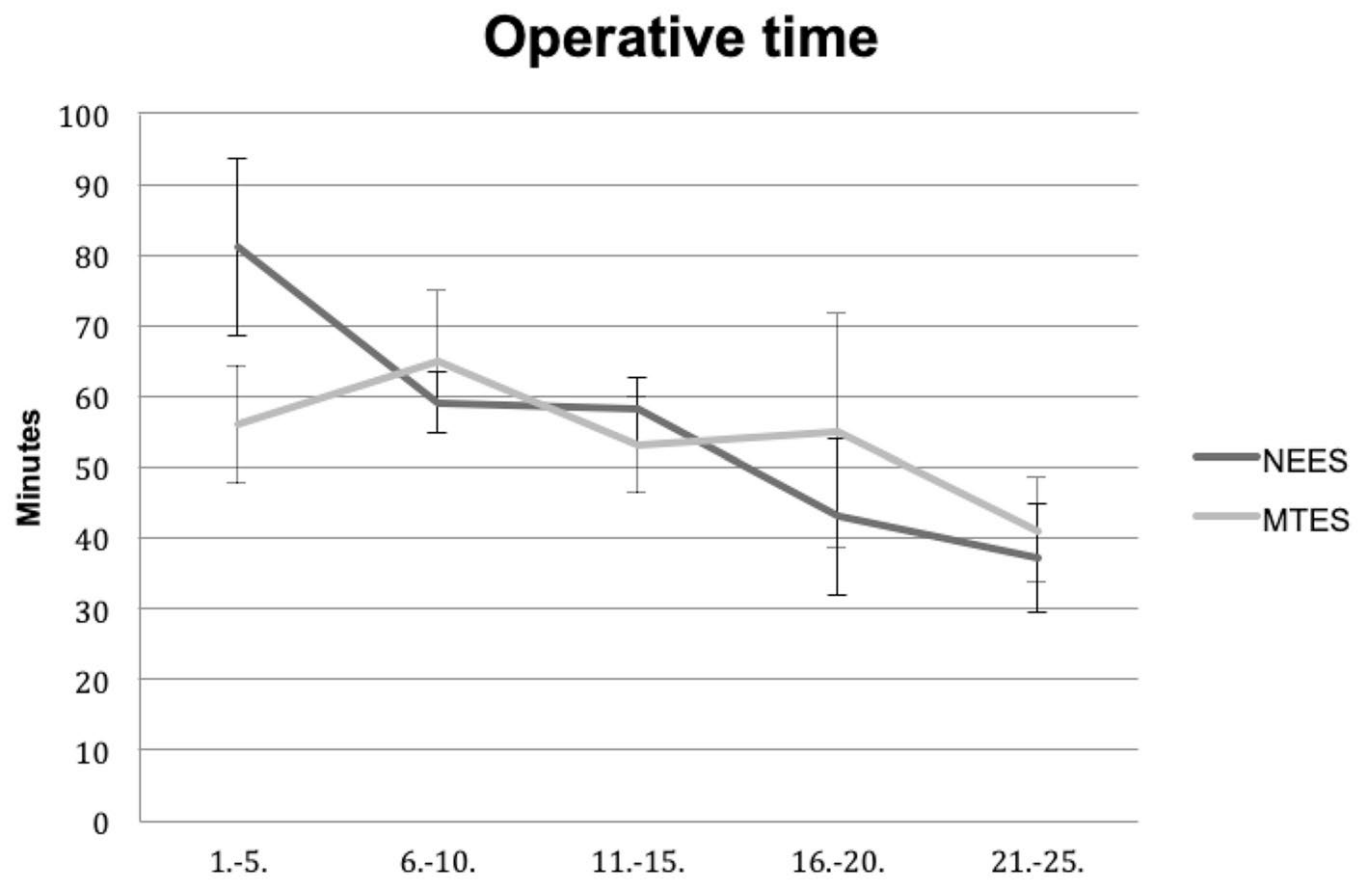
Mean duration of surgery in consecutive groups of 5 cases between microscopically trained ear surgeon (MTES) and native endoscopic ear surgeon (NEES)

**Fig. 2:**
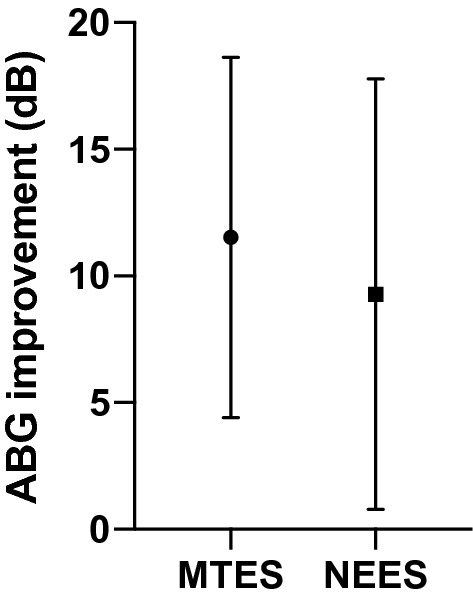
Postoperative improvement of ABG plotted as mean with standard deviation between MTES and NEES

Both surgeons achieved a reduction of the surgical time with increasing experience. The mean duration of surgery in the MTES and NEES groups was 54 ± 12.3 min and 55.6 ± 17.5 min, respectively. There was a significant improvement of the surgical time in the MTES and NEES group from the first five to the last five cases (Fig. 1). However, there was no significant difference between the MTES and NEES group for the mean surgical time overall and between the last five cases. Nevertheless, the surgical time of the first five operations in the MTES group was significantly shorter than in the NEES group. The GIR was 88% after 1 month and 92% after 3 months in both groups. Complications in the NEES group included two infections, one case of tinnitus and three cases of vertigo compared to two infections in the MTES group after 1 month. After 3 months there were two cases of tinnitus in the NEES group and two persistent infections in the MTES group (Table 2). Preoperative and postoperative PTA revealed a mean ABG-improvement of 11.5 ± 7.1 dB in MTES group versus 9.3 ± 8.5 dB in NEES group (Fig. 2). There was a significant improvement of the preoperative compared to the postoperative ABG for MTES und NEES. However, the difference of ABG improvement between MTES and NEES group was not statistically significant. Complete closure defined as postoperative ABG < 10 dB in MTES and NEES group was achieved in 52% and 64%, respectively. Air conduction thresholds for 6000 and 8000 Hz revealed no significant differences between the pre- and post-operative values for each group and between the NEES and MTES group (NEES: preoperative 41.8 ± 25.0 dB, postoperative 40.4 ± 27.4 dB; MTES: preoperative 39.4 ± 18.6 dB, postoperative 37.4 ± 17.2 dB). Detailed postoperative audiological results are presented in Table 2.

**Table 2:** Postoperative results

	MTES	NEES
Duration ± SD (min)	54 ± 12.3	55.6 ± 17.5
1 month		
GIR	88%	88%
Infection	2 (8%)	2 (8%)
Tinnitus	0	1 (4%)
Vertigo	0	3 (12%)
3 months		
GIR	92%	92%
Infection	2 (8%)	0
Tinnitus	0	2 (8%)
Vertigo	0	0
Hearing results		
Mean postoperative ABG ± SD (dB)	9.6 ± 6.5	8.1 ± 3.6
Mean improvement of ABG ± SD (dB)	11.5 ± 7.1	9.3 ± 8.5
Complete closure of ABG	13 (52%)	16 (64%)

## Discussion

The study compares the first 25 consecutive cases of type I tympanoplasty of an experienced microscopic-trained ear surgeon with those of an endoscopic-native ear surgeon. There was no need to convert to a microscopic approach in any case. The GIR and audiological results revealed similar results without statistically significant differences between the two groups. These results suggest analogous results for beginning endoscopic ear surgeons regardless of previous microscopic experience in tympanoplasty type I.

However, the first five cases were significantly shorter in the MTES compared to the NEES group. Apart from this, learning curves between the two surgeons seem similar with a statistically significant reduction of the surgical time in comparison with the first five cases to the last five cases. The difference in the beginning may reflect the 8 years of previous microscopic experience as well as the development of two-handed surgical skills. This might also explain the increased complication rate with tinnitus and vertigo of the native endoscopic ear surgeon as the one-handed technique can result in increased mechanical manipulation of the delicate middle ear structures in the beginning. Nevertheless, cochlear damage caused by one-handed manipulation or heat generation could recently not be objectified in comparison between microscopic and endoscopic tympanoplasties [8].

Compared to the literature different learning curves for microscopic and endoscopic approach to type I tympanoplasty have been described: using the microscopic technique, the learning curve reached a plateau for the operating time (78 min) and GIR (96%) after 29 cases [9]. In contrast, for the endoscopic type I tympanoplasty a surgeon with experience of approximately 50 microscopic tympanoplasty needed 50 endoscopic cases to gain a comparable result with GIR of 95% and operating time of 72 min [10]. To stabilize the operating time under 60 min a total of 150 cases were necessary in this study. In contrast, Dogan et al. [11] reported that an already microscopically trained surgeon needed only 60 endoscopic cases to achieve an operation time under 60 min. In terms of GIR even endoscopically native surgeons can achieve a rate of 90% in the beginning of otologic surgery [12]. This is comparable to the results in our study with a GIR of 92% after 3 months. However, our study shows a considerable shorter surgical time after only 25 consecutive cases for both surgeons. This could be because other endoscopic ear surgeries such as ossiculoplasty or cholesteatoma surgery were performed in the meantime (in between the type I tympanoplasties) and were excluded in this study.

Apart from the personally varying learning curves, endoscopic tympanoplasty differs technically most from microscopic tympanoplasty in offering better visualization at the expense of only one-handed and two-dimensional working possibilities [13]. This one-handed surgery might explain the initially longer operating time for the initial steps in endoscopic approaches. However, it has recently been suggested, that the endoscopic approach seems to be beneficial in teaching basic ear surgery skills. Especially inexperienced surgeons benefit significantly by learning the endoscopic technique for basic ear surgery skills first [14]. Unlike microscopically-trained surgeons, the mental model of endoscopic native surgeons is not yet consolidated. Similar to the literature regarding 3D endoscopy [15] the implementation of a relatively new technique by young surgeons may be facilitated by the more flexible mental representation of the technique. In contrast, microscopically experienced surgeons have to transform their fixed two-handed stereoscopic mental model into a one-handed two-dimensional model. This could explain the lack of significant advantages regarding surgical outcome and learning curve in the microscopically trained surgeon compared to the endoscopic native surgeon. Moreover, this could also serve as an explanation for necessary conversions during the first endoscopic tympanoplasties for microscopically trained surgeons as described in the literature [7], although this was not the case for any surgeon in this study. Nevertheless, learning the microscopic technique is essential for any ear surgeon in training, especially for the treatment of mastoid pathologies and for mastering potential complications of endoscopic surgery that require a microscopic approach.

Anyway, before performing endoscopic tympanoplasties on patients, gradual training has to be performed with utmost care in any case. In our opinion, this includes, first practicing on cadaveric human temporal bones, before performing the operation under appropriate supervision and guidance on humans systematically. Both surgeons attended such a training program, participated in otologic surgery courses and completed a fellowship in otology before their first endoscopic tympanoplasties. Apart from the utility of otologic training models, it is important to remember that not all surgical steps can be properly practiced there. Especially one-handed bleeding control in endoscopic ear surgery, which is unusual for beginners, can be challenging, but is safe and allows sufficient hemostasis [16, 17].

According to the literature further advantages of EES are the lack of retroauricular skin incision due to the transcanal endoscopic approach [13] as well as better postoperative health status and lower postoperative pain scores compared to microscopic tympanoplasty [18]. It seems that also anterior perforations, which often require a retroauricular approach with the microscopic technique, can be treated by transcanal endoscopic tympanoplasty type I [19]. Together with shorter operating times and less necessary equipment, the endoscopic approach might be also advantageous from a socio-economic point-of-view [12].

Type I tympanoplasty may be considered the ideal operation to start with ear surgery independently of the technique used. Of course, a thorough theoretical and practical training as e.g. taught during dissection courses is mandatory before starting to operate on patients. From our experience and from the results revealed in the present study we recommend the concomitant use of both endoscopic and microscopic technique during the education of residents and future ear surgeons. As described above, both techniques have advantages and challenges, and should therefore be used concomitantly to provide the best treatment to our patients. In our opinion the sequential teaching (first microscope, then endoscope) is not required as similar GIR and audiological results as well as an appropriate learning curve for EES were achieved. Therefore, we encourage direct application of endoscopic technique in type I tympanoplasties after thorough preparation and under appropriate supervision.

## Conclusion

Comparison of the first consecutive endoscopic type I tympanoplasties performed by a microscopically trained ear surgeon and a native endoscopic ear surgeon revealed comparable graft intake rate and audiological results. The learning curves between the two surgeons seem similar with a statistically significant reduction of the surgical time. We therefore recommend the parallel learning of both techniques and not sequential training.

## Data Availability

All data and materials can be made available upon request.

## Code availability

GraphPad Prism 8.
